# Association of Anemia with Cognitive Function and Dementia Among Older Adults: The Role of Inflammation

**DOI:** 10.3233/JAD-230483

**Published:** 2023-10-24

**Authors:** Jiao Wang, Chun Wang, Xuan Li, Jie Guo, Abigail Dove, Zhuang Cui, Weili Xu

**Affiliations:** a Department of Epidemiology and Biostatistics, School of Public Health, Tianjin Medical University, Tianjin, China; b Department of Epidemiology, College of Preventive Medicine, The Army Medical University (Third Military Medical University), Chongqing, China; c Tianjin Key Laboratory of Environment, Nutrition and Public Health, Tianjin, China; d Department of Neurobiology, Care Sciences and Society, Karolinska Institutet, Stockholm, Sweden

**Keywords:** Alzheimer’s disease, anemia, cognitive function, dementia, inflammation, UK Biobank

## Abstract

**Background:**

The association of anemia with cognitive function and dementia remains unclear.

**Objective:**

We aimed to investigate the association of anemia with cognitive function and dementia risk and to explore the role of inflammation in these associations.

**Methods:**

Within the UK Biobank, 207,203 dementia-free participants aged 60+ were followed for up to 16 years. Hemoglobin (HGB) and C-creative protein (CRP) were measured from blood samples taken at baseline. Anemia was defined as HGB <13 g/dL for males and <12 g/dL for females. Inflammation was categorized as low or high according to the median CRP level (1.50 mg/L). A subset of 18,211 participants underwent cognitive assessments (including global and domain-specific cognitive). Data were analyzed using linear mixed-effects model, Cox regression, and Laplace regression.

**Results:**

Anemia was associated with faster declines in global cognition (β= –0.08, 95% confidence interval [CI]: –0.14, –0.01) and processing speed (β= –0.10, 95% CI: –0.19, –0.01). During the follow-up of 9.76 years (interquartile range 7.55 to 11.39), 6,272 developed dementia. The hazard ratio of dementia was 1.57 (95% CI: 1.38, 1.78) for people with anemia, and anemia accelerated dementia onset by 1.53 (95% CI: 1.08, 1.97) years. The risk of dementia tended to be higher in people with both anemia and high CRP (1.89, 95% CI: 1.60, 2.22). There was a statistically significant interaction between anemia and CRP on dementia risk (p-interaction = 0.032).

**Conclusions:**

Anemia is associated with cognitive decline (specifically for processing speed) and increased risk of dementia, especially in people with high inflammation.

## INTRODUCTION

Anemia is a common clinical hematologic abnormality characterized by a decrease in hemoglobin concentration, red blood cell count, or filled cell volume, which is diagnosed by the patient’s hemoglobin concentration [1]. The incidence of anemia increases with age. About 17% of older adults have anemia [2]. Moreover, anemia has been related to higher risk of falls [3], disability [4], and mortality [5], as well as poorer quality of life [6] among older adults.

Previous studies have also associated anemia with poor cognitive function [7–13] and increased risk of dementia [14–17], but with some inconsistent findings [18–20]. Several observational studies have reported an association between anemia and poor global and domain-specific cognitive performance among older adults [7–10], but others have found no association [20]. Moreover, several studies failed to find a significant association between anemia and increased risk of dementia [19, 20]. In addition, most of these studies were cross-sectional and had a limited sample size, which restricted the statistical power of their findings. Therefore, studies with a longitudinal design and a large sample size are warranted to explore the associations of anemia with the long-term performance of cognitive function and risk of incident dementia.

Moreover, anemia and inflammation may share biological mechanisms and have similar manifestations like increased levels of several proinflammatory mediators [21]. Elevated inflammation is a risk factor for cognitive impairment [22] and dementia [23, 24]. Furthermore, growing evidence suggests that older adults with chronic conditions have poorer cognitive function and a higher risk of dementia [25], and anemia often appears as a marker of chronic conditions. Inflammation and chronic conditions may play a modulating role in the association between anemia and an increased risk of dementia. However, to the best of our knowledge, no studies have investigated the role of inflammation/chronic conditions in the associations between anemia and cognitive performance and dementia.

We hypothesize that anemia is associated with faster decline in cognitive function and a higher risk of dementia, and further that high levels of inflammation or the presence of chronic conditions may exacerbate the anemia-dementia association. In the present study, we aimed to verify these hypotheses by 1) examining the association of anemia with cognitive function change and risk of dementia; and 2) exploring the joint effects of anemia and inflammation/chronic conditions on dementia using data from the UK Biobank.

## METHODS

### Study design, setting, and participants

**Fig. 1:**
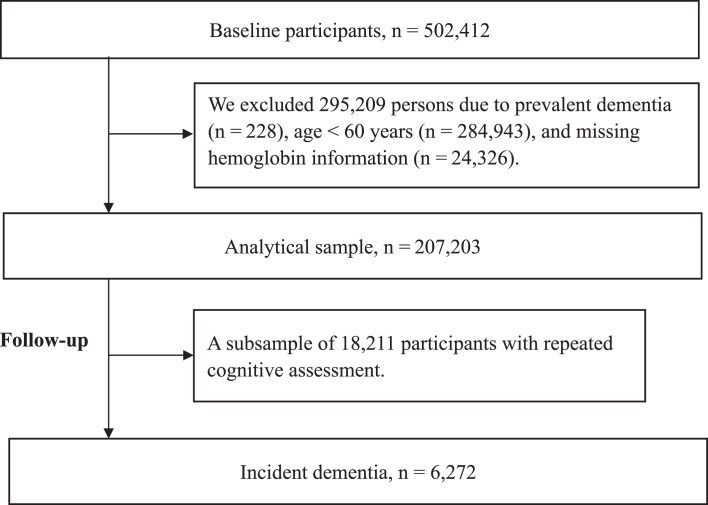
Flowchart of the study population.

Data were obtained from the UK Biobank, a large-scale population-based cohort of 502,412 UK residents ranging from 7 to 73 years of age at baseline [26]. After excluding participants with prevalent dementia at baseline (*n* = 228), aged <60 years (*n* = 284,943), or missing data for hemoglobin (*n* = 1,323), 207,203 participants remained in the current study, 18,211 of whom had repeated cognitive assessments during follow-up (with ≥1 waves of follow-up) (Fig. 1).

The UK Biobank study received ethical approval from the North West Multi-Centre Research Ethics Committee (21/NW/0157) and all enrolled participants provided informed and written consent.

### Data collection

Information on demographic characteristics, socioeconomic status, and lifestyle factors was collected at baseline through a computerized touchscreen questionnaire. A comprehensive physical measurement and clinical evaluation were performed on the enrolled subjects at baseline, and extensive cognitive tests were administered at baseline and during the follow-up. (Supplementary Table 1).

Ethnic background was dichotomized as White versus non-White. Socioeconomic status was assessed by the Townsend Deprivation Index (TDI). Education was dichotomized as college or non-college. Height and weight were measured at baseline while participants were wearing light clothing and no shoes. Body mass index (BMI) was calculated as weight (kg)/height (m^2^). Smoking and alcohol status were categorized as never, former, or current smoker/drinker. Regular physical activity was operationalized as at least 150 min of moderate activity per week, 75 min of vigorous activity per week, or an equivalent combination. Social connection was assessed by the following question: “How often do you visit friends or family or have them visit you?” Response options were as follows: Almost daily/2–4 times a week/About once a week/About once a month/Once every few months/Never or almost never/No friends/family outside household/Do not know/Prefer not to answer. These responses were further classified as active or inactive according to the median. Moreover, the *apolipoprotein E* (*APOE*) gene was genotyped and further stratified as carriers versus non-carriers of the *ɛ*4 allele.

Serum C-creative protein (CRP) level (mg/L) was measured by immunoturbidimetric high-sensitivity analysis on the Beckman Coulter AU5800 analytical platform (Beckman Coulter Inc., Brea, CA, USA) [27]. The manufacturer’s analytical range was 0.08–79.96 mg/L. Further information is available from official UK Biobank documentation on CRP specifically [28] and the companion document for biomarker data [29]. Participants were dichotomized as having a low or high CRP level groups based on the median value (i.e., CRP = 1.50 mg/L). Chronic diseases were determined using primary and secondary diagnoses from the International Classification of Diseases, Ninth and Tenth Revisions (ICD 9 and 10), hospital admission data from England, Scotland, and Wales, and cancer registry data [30]. ICD codes were classified into 12 chronic disease subgroups by ICD chapter (Supplementary Table 2). Chronic conditions were defined as the presence of any of these 12 chronic disease subgroups and categorized as the presence versus absence. Estimated glomerular filtration rate (eGFR, mL/min/1.73 m^2^) was calculated using the Chronic Kidney Disease Epidemiology Collaboration (CKD-EPI) 2012 formula with creatinine and Cystatin C and was categorized as normal (>60 mL/min/1.73 m^2^), or impaired (<60 mL/min/1.73 m^2^).

### Assessment of anemia

Blood was collected into a 4-mL EDTA vacutainer and dispatched to the central processing laboratory in temperature-controlled shipping boxes (at 4°C) [31]. Samples were typically analyzed using a Coulter counter (Beckman Coulter) at the UK Biobank central laboratory within 24 h of the blood draw. Hemoglobin concentration (HGB) was directly measured with the Coulter method using the LH750 Haematology Analyser. Anemia was defined as HGB <13 g/dl for men and <12 g/dl for women according to the World Health Organization (WHO) criteria; participants were dichotomized as anemia versus anemia-free [32].

### Assessment of cognitive function

Cognitive function was assessed at baseline and each follow-up visit based on five tests administered through a touchscreen interface [33], including the working memory test (the longest numeric string correctly recalled), prospective memory test (successfully carrying out instruction after a filled delay), visual memory test (the number of errors when recalling positions of matching cards), verbal reasoning test (the number of correct answers to logic/reasoning-type questions), and processing speed test (mean time to correctly match cards). Results of the numerical memory and visual memory tests were reverse-coded to allow higher scores to reflect better performance. Raw scores for each cognitive test were converted to z-scores and then averaged to yield a composite score for global cognitive function [34].

### Assessment of dementia

Dementia was ascertained based on algorithmically-defined outcomes obtained through combinations of coded information from self-report (Data-Field 20002, code: 1263), medical records (ICD-10 codes: F00, F01, F02, F03, F05.1, G30, G31.1, G31.8), and death records (ICD-10 codes: F00, F01, F02, F03, F05.1, G30, G31.1, G31.8) from the Hospital Episode Statistics (England), the Scottish Morbidity Record (Scotland), and the Patient Episode Database (Wales). The date of incident dementia was set as the earliest date of dementia diagnosis recorded regardless of the source used.

### Statistical analysis

Baseline characteristics of the study population by anemia status were analyzed using Chi-square tests for categorical variables and *t*-test or Wilcoxon rank-sum tests for continuous variables.

Linear mixed-effects models were used to estimate the associations of anemia with global cognition and domain-specific cognitive function. The fixed effect included anemia, follow-up time, and their interaction term. The random effect included random intercept and slope, allowing the individual differences at baseline and across follow-up. The Cox proportional hazards regression models were used to estimate the hazard ratios (HRs) and 95% CIs for the incidence of dementia associated with anemia. Laplace regression models were used to estimate the differences in median time (years) and 95% CIs of dementia onset between anemia and non-anemia group. Follow-up time was calculated as the time from study entry to dementia diagnosis, death, or the final examination, whichever occurred first. The models were adjusted for age, sex, and education (basic models) and then further adjusted for race, TDI, smoking status, alcohol status, BMI, physical activity, social connection, *APOE* *ɛ*4, CRP, and chronic conditions (multivariable-adjusted models). We also repeated the analyses after stratifying by gender. The joint effects of anemia with CRP and chronic conditions on dementia were assessed by combining anemia (with versus without) and CRP (high versus low level) or chronic conditions (yes versus no). Statistical interaction was examined by establishing an indicator variable with the cross-product of two variables.

In sensitivity analysis, we repeated the analyses after 1) using multiple imputation to handle covariates with missing values (*n* = 34,574), 2) further adjusted for eGFR, 3) stratifying by smoking status, physical activity level, and eGFR, 4) using the competing risks model (the Fine-Gray model) with death as the competing event, and 5) excluding participants with incident dementia that occurred during the first 5 years of follow-up (*n* = 586). Alpha was set at 0.05 for all analyses. All statistical analyses were performed using Stata SE 16.0 (Stata Corp, College Station, TX, USA).

## RESULTS

### Baseline characteristics

Of the 207,203 participants (mean age [standard deviation] = 64.13 [2.85] years, 52.52% female), 8,209 (3.96%) had anemia. Compared to participants without anemia, those with anemia were older, more likely to be non-White, more likely to have a lower socioeconomic status, education, and BMI, less likely to be current alcohol drinkers, current smokers, and engage in physical activity, and more likely to have active social connection, high CRP levels, and chronic conditions (*p* < 0.05 for all) (Table 1). There were no significant differences in sex or APOE *ɛ*4 carrier status between the two groups.

**Table 1:** Characteristics of the study population by anemia (*n* = 207,203)

Characteristics	Anemia (*n* = 8,209)	Non-anemia (*n* = 198,994)	*p*
Age (y)	64.61±2.90	64.11±2.85	<0.001
Female	4,285 (52.20)	104,536 (52.53)	0.553
Race-White	7,382 (90.47)	185,407 (93.60)	<0.001
Townsend deprivation index	–1.66 (–3.38, 1.34)	–2.39 (–3.76, 0.01)	<0.001
Education (college)	1,760 (21.91)	51,914 (26.47)	<0.001
BMI (kg/m^2^)	27.76±5.36	27.58±4.50	0.003
Alcohol consumption status			<0.001
Never	722 (8.83)	9,139 (4.60)
Former drinking	655 (8.01)	7,198 (3.62)
Current drinking	6,798 (83.16)	182,248 (91.77)
Smoking status			<0.001
Never	3,876 (47.61)	98,994 (50.05)
Former smoker	3,729 (45.81)	82,387 (41.65)
Current smoker	536 (6.58)	16,423 (8.30)
Regular physical activity	4,464 (63.80)	128,100 (71.64)	<0.001
Active social connection	1,281 (15.79)	28,571 (14.46)	<0.001
C-reactive protein	1.94 (0.86, 4.69)	1.49 (0.77, 2.97)	<0.001
*APOE* *ɛ*4 carrier	1,963 (28.49)	46,928 (28.31)	0.751
Chronic conditions	5,253 (63.99)	92,263 (46.36)	<0.001

Values are mean±SD, *n* (%), or median (interquartile range). *APOE* *ɛ*4, *apolipoprotein E* epsilon 4; BMI, body mass index. Missing data: Education = 3,010; Race = 958; Smoking = 1,258; Alcohol consumption = 443; Regular physical activity = 21,394; Active social connection = 1,476; *APOE* *ɛ*4 = 34,574.

### Association between anemia and cognitive function

In multivariable-adjusted mixed-effects models, participants with anemia had faster declines in global cognition and processing speed than those without anemia (Table 2). After stratifying by gender, the association between anemia and the changes in global cognitive function performance was similar among males and females [β [95% CI]: –0.11 [–0.20, –0.02]/ –0.03 [–0.12, –0.53]. However, the associations between anemia and changes in processing speed were not statistically significant in neither females nor males (Table 2).

**Table 2:** β coefficient and 95% confidence intervals (CIs) of long-term performance in global and domain-specific cognitive functions in relation to anemia

Anemia	Global cognition β (95% CI)^†^	Working memory β (95% CI)^†^	Prospective memory β (95% CI)^†^	Visual memory β (95% CI)^†^	Verbal reasoning β (95% CI)^†^	Processing speed β (95% CI)^†^
Total population
No	Reference	Reference	Reference	Reference	Reference	Reference
Yes	–0.08 (–0.14, –0.01) ^†^	0.19 (–0.11, 0.48)	–0.10 (–0.25, 0.06)	–0.04 (–0.14, 0.05)	0.06 (–0.08, 0.19)	–0.10 (–0.19, –0.01)^†^
Female
No	Reference	Reference	Reference	Reference	Reference	Reference
Yes	–0.03	0.15	–0.04	–0.05	0.02	–0.08
	(–0.12, –0.53)^†^	(–0.26, 0.56)	(–0.26, 0.18)	(–0.08, 0.18)	(–0.16, 0.20)	(–0.20, 0.04)
Male
No	Reference	Reference	Reference	Reference	Reference	Reference
Yes	–0.11	0.18	–0.18	–0.13	0.11	–0.06
	(–0.20, –0.02)^†^	(–0.26, 0.61)	(–0.41, 0.05)	(–0.28, 0.01)	(–0.09, 0.32)	(–0.18, 0.06)

^†^ Model adjusted for age, sex, education, race, Townsend deprivation index, smoking status, alcohol consumption status, body mass index, regular physical activity, active social connection, *apolipoprotein E* epsilon 4, C-creative protein, and chronic conditions. ^*^p < 0.05.

### Association between anemia and dementia

During the follow-up (median: 9.76 years, interquartile range [IQR]: 7.55 to 11.39 years), 6,272 participants developed dementia. Compared to participants without anemia, those with anemia had a higher risk of dementia in both basic- and multi-variable adjusted models (HR [95% CI]: 1.90 [1.72, 2.10]/1.57 [1.38, 1.78]) (Table 3). The association between anemia and increased risk of dementia was present in both males (HR [95% CI]: 1.75 [1.48, 2.07]) and females (HR [95% CI]: 1.38 [1.14, 1.68]). In addition, there was a significant multiplicative interaction between sex and anemia for increased risk of dementia (*p-interaction* = 0.048,Table 3).

**Table 3:** Hazard ratio (HRs), 50th percentile differences (PDs) in years, and 95% confidence intervals (CIs) of dementia in relation to anemia

Anemia	No. of subjects	No. of cases	Cox Regression		Laplace Regression	
		HR (95% CI)^†^	HR (95% CI)^‡^	50th PDs (95% CI)^†^	50th PDs (95% CI)^‡^
Total population
No	198,994	5,828	Reference (1.00)	Reference (1.00)	Reference (0)	Reference (0)
Yes	8,209	444	1.90 (1.72, 2.10)^*^	1.57 (1.38, 1.78)^*^	–0.18 (–0.22, –0.15)^*^	–1.53 (–1.97, –1.08)^*^
Female
No	104,536	2,780	Reference (1.00)	Reference (1.00)	Reference (0)	Reference (0)
Yes	4,285	195	1.67 (1.44, 1.94)^*^	1.38 (1.14, 1.68)^*^	–0.11 (–0.15, –0.07)^*^	–1.07 (–1.73, –0.42)^*^
Male
No	94,458	3,048	Reference (1.00)	Reference (1.00)	Reference (0)	Reference (0)
Yes	3,924	249	2.12 (1.86, 2.42)^*^	1.75 (1.48 2.07)^*^	–0.32 (–0.38, –0.26)^*^	–1.93 (–2.53, –1.33)^*^

^†^Adjusted for age, sex, and education. ^‡^Adjusted for age, sex, education, race, Townsend deprivation index, smoking status, alcohol consumption status, body mass index, regular physical activity, active social connection, *apolipoprotein E* epsilon 4, C-creative protein, and chronic conditions. ^*^p < 0.05. The interaction between anemia and sex on incident dementia is significant (p-interaction = 0.048).

The median onset age of dementia was 74.52 (95% CI: 71.59–77.74) years for participants with anemia and 74.94 (95% CI: 72.42–77.74) years for those without anemia. Compared to the anemia-free group, the median onset of dementia in the anemia group was 1.53 (1.08, 1.97) years earlier (Table 3).

### Joint effect of anemia and CRP/chronic conditions on dementia risk

**Fig. 2:**
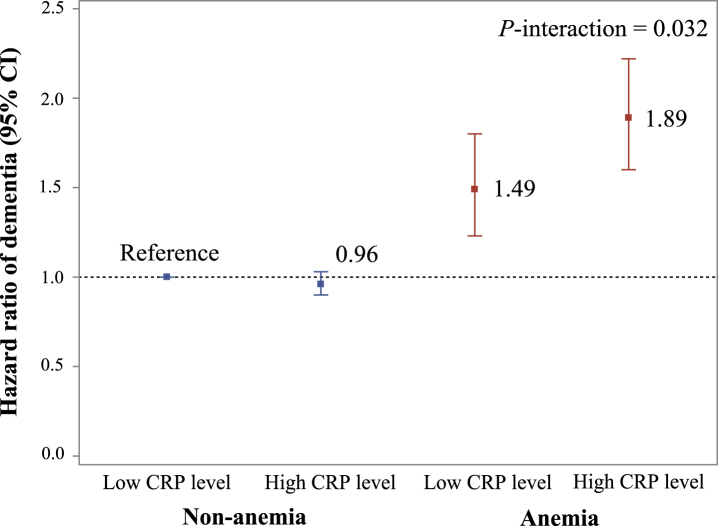
Joint effect of anemia and CRP level on risk of dementia. Model adjusted for age, sex, education, race, Townsend deprivation index, smoking status, alcohol consumption status, body mass index, regular physical activity, active social connection, apolipoprotein E epsilon 4, and chronic conditions. p-interaction <0.05 refers to a significant multiplicative interaction between anemia status and the level of C-creative protein on dementia. There is not significant difference between anemia with low CRP level and anemia with high CRP level (*p* = 0.058).

In the joint effect analyses, compared to anemia-free participants with low CRP, the HR of dementia was 1.89 (95% CI 1.60, 2.22) among people with anemia and high CRP and 1.49 (1.23, 1.80) among those with anemia and low CRP (Fig. 2 and Supplementary Table 3). There was a significant multiplicative interaction between anemia and high CRP on dementia risk (*p-interaction* = 0.032). Compared to anemia-free, chronic disease-free participants, the HR of dementia was 2.57 (2.21, 2.98) among people with anemia and chronic conditions and 1.54 (1.21, 1.95) among those with anemia and no chronic conditions. No such interaction was present for anemia and chronic conditions (*p-interaction* = 0.290).

### Sensitivity analysis

In sensitivity analysis, the results were not much altered when we repeated the analysis after 1) performing multiple imputation to account for missing values of some covariates (including education, race, smoking, alcohol consumption, regular physical activity, and regular social contact; Supplementary Table 4), 2) further adjusted for eGFR (Supplementary Table 5), 3) stratifying by smoking status, physical activity, and eGFR (Supplementary Tables 6–8), 3) taking into account the competing risk of death using Fine and Gray regression (Supplementary Table 8), and 4) excluding 586 participants who developed dementia within the first 5 years of follow-up (Supplementary Table 9).

## DISCUSSION

In this large prospective cohort study from the UK Biobank, we found that 1) anemia was associated with faster declines in global cognitive function and processing speed; 2) anemia was related to a higher risk of dementia in both females and males, and can anticipate the onset of dementia by 1.53 years; and 3) a high level of inflammation appears to exacerbate the association of anemia and dementia.

Limited observational studies have linked anemia to poor cognitive performance [7–13, 20] and increased risk of dementia [14–17]. A number of cross-sectional studies have shown that anemia is associated with poor global cognitive function [7, 8, 20], with the exception of one cross-sectional study from the United States [18]. Only two cohort studies revealed that anemia was associated with poor global cognition [9, 10]. However, the association between anemia and domain-specific cognitive performance is unclear [11–13]. Anemia has been related to poor performance in immediate verbal recall in the Women’s Health and Aging Study II [13] and in episodic memory according to a telephone interview study of cognitive status among Chinese middle-aged and elderly [11]. However, one study found that anemia was not associated with word list recall or word list recognition [12]. There are four longitudinal studies consistent with our study showing that anemia was related to increased risk of dementia [14–17]. Although these prior studies provided evidence about the possible role of anemia in cognitive performance and dementia, most of them have small sample sizes (436 to 10,918) and relatively short follow-ups (4 to 5 years), resulting in poor generalization. In this study, we found that anemia was associated with poor performance on global cognition and increased risk of dementia in the long-term follow-up period. Most striking was anemia was associated with processing speed, this result has not previously been described.

Additionally, considering females have a slightly higher anemia prevalence than males, we conducted sex-stratified analyses. We observed that the association of anemia with poor global cognition and increased risk of dementia was similar in males and females. This is in contrast to the results of a previous study which indicated a higher level of cognitive decline in anemic females than males over 4 years of follow-up [15]. Further research is required to clarify the sex differences between anemia and dementia development.

The aging process is accompanied by alterations in the regulation of hematopoiesis and inflammation, making that anemia and high levels of inflammation common among older people [2]. Anemia and inflammation may be interrelated, as they have partially overlapping biological mechanisms [21]. In addition, both basic science and epidemiological studies have shown that inflammation plays a role in the pathogenesis of dementia [35]. However, to date, no studies have explored the joint effect of anemia and inflammation on the risk of dementia. In the current study, we found a significant joint effect between anemia and CRP, meaning that high levels of CRP may exacerbate the association between anemia and an increased risk of dementia. Our findings suggest that systemic interventions to reduce peripheral pro-inflammatory signals could be considered as a preventive strategy to slow the progression of dementia in older adults. It is well known that the accumulation of chronic conditions increases the complexity of the disease and can further impair physical health [36]. Chronic conditions have been linked to an increased risk of both anemia [37] and dementia [38]. Nevertheless, in the current study, there was no significant joint effect between anemia and chronic conditions on the risk of dementia, suggesting that the effect of anemia on dementia is independent from chronic conditions.

There are several mechanisms that could connect anemia to poor cognitive health. First, anemia may limit cerebral blood flow, leading to insufficient oxygen supply to the brain [39]. Emerging work suggests that prolonged hypoxia alters the excitability and functional expression of iron channels [40]. It escalates the process of amyloid-β protein formation through the amyloidogenic transformation of the amyloid-β protein precursor [41] thus contributing to poor cognition and incident dementia. Second, prolonged exposure to low-grade inflammation could drive neurodegeneration and poor cognition [42]. Proinflammatory cytokines related to inflammation could alter the expression and processing of amyloid-β protein precursor, indirectly causing poor cognition [43]. IL-1 also increases neuronal tau phosphorylation [44] and activates astrocytes [45]. Third, frailty is another consequence of anemia, as a marker of increased vulnerability, which is associated with mitochondrial dysfunction and an increased risk for poor cognition [46]. Reactive oxygen species are produced in excess due to mitochondrial dysfunction, and this excess synthesis causes oxidative damage to neurons, which lowers cognition [47]. Finally, it is also possible that poor cognition is a result of a shortage of B-vitamins (e.g., vitamin B12 or folic acid) related to anemia [48]. Evidence shows that folic acid and serum vitamin B12 deficiency are associated with neurodegenerative disease and cognitive impairment [49] by elevated homocysteine levels. Homocysteine elevation lead to increased oxidative stress [49], facilitates amyloid-β protein deposition [50], promote the atrophy of key brain regions related to the process of AD and cognitive decline [51].

This study’s strengths include the large sample size, relatively long follow-up period, and availability of data on demographics, medical history, and lifestyle. Nonetheless, some limitations need to be pointed out. First, the UK Biobank participants were volunteers likely healthier than the general population, which may lead to an underestimation of the association of anemia with poor cognitive performance and dementia. Furthermore, caution is required when generalizing our results to populations outside of white European ancestry. Second, since dementia was diagnosed using electronic health records data, some cases may have been missed, which may attenuate the relationship between anemia and the risk of dementia. Third, given the similar clinical manifestations of anemia and cognitive decline, it could be difficult to determine the chronological order of these two disorders. Thus, potential reverse causation could not be completely ruled out. To avoid the effect of possible undiagnosed dementia on the results, we excluded 586 participants who developed dementia during the first 5 years of follow-up, and the results did not change much. Fourth, it is impossible to completely rule out potential confounders caused by unmeasured exposure to toxins. Fifth, cognitive data from UKB were not collected annually, which makes it more difficult to explore cognitive changes, hence we only focused on long-term cognitive performance in this study. Finally, micronutrient profiles, iron intake, and serum ferritin as possible mechanisms of anemia may play a role in the association of anemia with cognitive decline and dementia, but the data are not available.

In conclusion, our study provides evidence that anemia is associated with poor cognitive performance and increases the risk of dementia. High levels of CRP may exacerbate the association between anemia and dementia. Our results emphasize the importance of early detection and prevention of anemia to slow down the progression of cognitive function in older adults.

## Supplementary Material

Supplementary Material

## Data Availability

The data supporting the findings of this study are openly available in UK Biobank at https://www.ukbiobank.ac.uk. These data were derived from the following resources available in the public domain: https://www.ukbiobank.ac.uk.
