# Supplementary material for: Association of Anemia with Cognitive Function and Dementia Among Older Adults: The Role of Inflammation
Source: J Alzheimers Dis. 2023 Oct 24;96(1):125–34. doi: 10.3233/JAD-230483 (PMC10657670; doi:10.3233/JAD-230483)
Supplement: Supplementary Material [file jad-96-jad230483-s001.pdf]

Supplementary Material

Association of Anemia with Cognitive Function and Dementia Among Older Adults: The Role of Inflammation

Supplementary Table 1. Characteristics of the study population by incident dementia status.

**Characteristics**	**Dementia (n =6,272)**	**Non-dementia (n =200,931)**	***p***
Age (y)	65.68 ± 2.71	64.09 ± 2.84	<0.001
Female	2,975 (47.43)	105,846 (52.68)	<0.001
Race-White	5,795 (92.99)	186,994 (93.49)	0.113
Townsend deprivation index	-1.05 ± 3.25	-1.57 ± 2.95	<0.001
Education (college)	1,235 (20.20)	52,439 (26.47)	<0.001
BMI (kg/m^2)	27.77 ± 4.83	27.58 ± 4.52	0.003
Alcohol consumption status			
Never	446 (7.14)	9,415 (4.70)	<0.001
Former drinking	411 (6.58)	7,442 (3.71)	<0.001
Current drinking	5,390 (86.28)	183,656 (91.59)	<0.001
Smoking status			
Never	2,835 (45.66)	100,035 (50.08)	<0.001
Former smoker	2,794 (45.00)	83,322 (41.72)	<0.001
Current smoker	580 (9.34)	16,379 (8.20)	<0.001
Regular physical activity	3,586 (68.21)	128,978 (71.44)	<0.001
Regular social contact	940 (15.18)	28,912 (14.49)	<0.001
C-reactive protein	2.88 ± 4.81	2.84 ± 4.67	0.573
APOE ε4 carrier	2,864 (54.80)	46,027 (27.49)	<0.001
Chronic conditions	3,743 (59.68)	93,773 (46.67)	<0.001
Death	3,114 (49.65)	22,308 (11.10)	<0.001
Anemia	444 (7.08)	7,765 (3.86)	<0.001

Values are mean ± SD, n (%), or median (interquartile range).
APOE ε4, *apolipoprotein E* epsilon 4; BMI, body mass index.
Missing data: Education=3,010; Race=958; Smoking=1,258; Alcohol consumption=443; Regular physical activity=21,394; Active social connection=1,476; *APOE* ε4 carrier=34,574.

Supplementary Table 2. ICD code for chronic conditions.

Disease	ICD-10 Code	ICD-9 Code
Infectious Disease		
Tuberculosis	A150 A151 A152 A153 A156 A157 A159 A160 A162 A163 A164 A165 A169 A170 A178 A180 A181 A182 A183 A184 A185 A187 A188 A190 A192 A199	01192 01199 0130 01619 1729 01789
Chronic Viral Hepatitis	B180 B181 B182 B188 B189	0703
HIV	B200 B201 B202 B203 B204 B206 B207 B208 B210 B211 B212 B213 B217 B218 B220 B221 B222 B227 B230 B232 B238 B24	
Malignant neoplasms		
Cervix	C530 C531 C538 C539	1800 1809
Ovary	C56	1830
Testis	C620 C621 C629	1860 1869
Lip, oral cavity, and pharynx	C01 C020 C021 C022 C023 C024 C028 C029 C030 C031 C039 C040 C041 C048 C049 C050 C051 C052 C058 C059 C060 C061 C062 C068 C069 C07 C080 C081 C088 C089 C090 C091 C098 C099 C100 C102 C103 C104 C108 C109 C110 C111 C112 C113 C119 C12 C130 C131 C138 C139 C140 C148	1413 1416 1419 1420 1440 1460
Esophagus	C150 C151 C152 C153 C154 C155 C158 C159	1505
Stomach	C160 C161 C162 C163 C164 C165 C166 C168 C169	1519
Small Intestine	C170 C171 C172 C173 C178 C179	1521
Colon rectum and anus	C180 C181 C182 C183 C184 C185 C186 C187 C188 C189 C19 C210 C211 C218	1530 1532 1533 1534 1536 1537 1539 1540 1541 1542 1543
Liver and intrahepatic bile ducts	C220 C221 C223 C224 C227 C229	1551
Gallbladder & biliary tract	C23 C240 C241 C248 C249	1561
Pancreas	C250 C251 C252 C253 C254 C257 C258 C259	1570 1574 1579
Ill-defined digestive organs	C260 C261 C268 C269	1590
Sinuses, larynx, & trachea	C310 C311 C312 C313 C319 C320 C321 C322 C323 C328 C329 C33	1600 1610 1613 1619
Bronchus & lung	C340 C341 C342 C343 C348 C349	1623 1629
Ill-defined sites in the respiratory system and intrathoracic organs	C300 C301 C390 C384 C383 C382 C381 C380 C37	1649
Bone & articular cartilage	C400 C401 C402 C403 C408 C410 C411 C412 C413 C414 C419	1702 1707 1709
Melanoma	C430 C431 C432 C433 C434 C435 C436 C437 C438 C439	1720 1723 1725 1726 1727 1729
Other skin	C440 C441 C442 C443 C444 C445 C446 C447 C448 C449	1730 1731 1732 1733 1734 1735 1736 1737
Mesothelioma	C450 C451 C457 C459 C440	
Other Neoplasms	C460 C463 C467 C469 C471 C472 C473 C475 C476 C479 C480 C481 C482 C488 C490 C491 C492 C493 C494 C495 C496 C498 C499 C510 C511 C518 C519 C52 C570 C571 C574 C577 C578 C579 C58 C600 C601 C602 C608 C609	1950 1951

	C631 C632 C637 C639 C760 C761 C762 C763 C764 C765 C767 C768 C800 C809 C97	
Breast	C500 C501 C502 C503 C504 C505 C506 C508 C509	1740 1743 1744 1745 1748 1749
Uterus	C540 C541 C542 C543 C548 C549 C55	1799
Prostate	C61	1859
Kidney, except renal pelvis	C64	1890
Renal pelvis & ureter	C65 C66	
Bladder	C670 C671 C672 C673 C674 C675 C676 C677 C678 C679	1882 1889
Unspecified urinary organs	C680 C688 C689	
CNS	C690 C691 C692 C693 C694 C695 C696 C698 C699 C700 C701 C709 C710 C711 C712 C713 C714 C715 C716 C717 C718 C719 C720 C721 C722 C724 C725 C729	1906 1909 1913 1916 1919
Endocrine gland	C73 C740 C741 C749 C750 C751 C755 C759	1939
Lymphoid, hematopoietic, and related tissue	C810 C811 C812 C813 C814 C817 C819 C820 C821 C822 C823 C824 C826 C827 C829 C830 C831 C832 C833 C834 C835 C836 C837 C838 C839 C840 C841 C843 C844 C845 C846 C847 C848 C849 C850 C851 C852 C857 C859 C860 C862 C863 C865 C866 C880 C883 C884 C900 C901 C902 C903 C910 C911 C912 C913 C914 C915 C916 C917 C918 C919 C920 C921 C922 C923 C924 C925 C926 C927 C928 C929 C930 C931 C940 C942 C944 C945 C946 C947 C950 C951 C959 C961 C962 C963 C964 C966 C967 C969	2001 2015 2016 2017 2019 2020 2024 2028 2029 2040 2050 2051 2059
Diseases of the blood and blood-forming organs and certain disorders involving the immune mechanism		
Nutritional anemias	B500 B501 B508 B509 B510 B513 B518 B519 B529 B530 B531 B538 B539	28099
Hemolytic anemias	B550 B552 B561 B563 B569 B570 B571 B572 B573 B578 B580 B581 B582 B588 B589 B590 B591 B592 B593 B594 B595 B598 B599	2820 2829 2830
Aplastic anemias	D609 D610 D611 D612 D613 D619	2849
Anemia of chronic disease	D630 D638	
Coagulation defects	D66 D67 D680 D681 D682 D683 D684 D685 D686 D688 D689	2860 2864 2869
Platelet Disorders	D690 D691 D692 D693 D694 D695 D696	2870 2871 2872 28730 28739 2875
Immunodeficiency disorders excluding HIV	D70 D800 D801 D802 D803 D804 D805 D806 D808 D809 D810 D811 D818 D819 D821 D823 D824 D829 D830 D831 D832 D838 D839 D841 D848 D849	
Sarcoid	D860 D861 D862 D863 D868 D869	1359
Endocrine, nutritional, and metabolic diseases		
Hypothyroidism	E011 E030 E032 E034 E038 E039	24499
Non-toxic goiter	E040 E041 E042 E048 E049	2410 2411 2419
Thyrotoxicosis	E050 E051 E052 E053 E055 E058 E059	2420 2422 2423 2429

Insulin dependent diabetes mellitus	E100 E101 E102 E103 E104 E105 E106 E107 E108 E109	25010 25011 25019
Non-insulin dependent diabetes mellitus	E110 E111 E112 E113 E114 E115 E116 E117 E118 E119	
Unspecified diabetes mellitus	E140 E142 E143 E144 E145 E146 E147 E148 E149	25000 25001 25009 25029 2503 2504 2505 25099
Hyperparathyroidism	E210 E211 E212 E213	2520
Acromegaly & Gigantism	E220	2530
Hypopituitarism	E230	25329
Cushings Syndrome	E240 E241 E242 E248 E249	2550
Adrenocortical insufficiency	E271 E272 E273 E274	25549
Disorders of lipoprotein metabolism	E780 E781 E782 E784 E785 E789	2722 27249
Mental Health		
Mental and behavioral disorders due to use of alcohol	F100 F101 F102 F103 F104 F105 F106 F107 F108 F109	2910 2918
Bipolar disorder	F310 F311 F312 F313 F314 F315 F316 F317 F318 F319	2961
Depressive disorder	F320 F321 F322 F323 F328 F329 F330 F331 F332 F333 F334 F338 F339	3119
Anxiety Disorders	F400 F401 F402 F408 F410 F411 F412 F413 F418 F419	3000
Reaction to severe stress, and adjustment disorders	F430 F431 F432 F438 F439	
Diseases of the nervous system		
Chronic suppurative neurological disease	G060 G061 G062	
Systemic atrophies primarily affecting the central nervous system	G10 G111 G112 G114 G118 G119 G121 G122 G129 G130 G131 G14	
Extrapyramidal and movement disorders	G20 G210 G211 G214 G218 G219 G231 G238 G258 G259	3320 3321 3335 3336 33379 3338 3339
Other degenerative diseases of CNS	G300 G301 G308 G309 G310 G312 G318 G319	
Multiple sclerosis	G35	3409
Other demyelinating diseases of central nervous system	G360 G369 G371 G372 G373 G378 G379	3418 3419
Epilepsy	G403 G404 G406 G407 G408 G409	34510 34519 34550 34559 3459
Migraine	G430 G431 G432 G433 G438 G439	3460 3461 3462 3468 3469
TIA	G450 G451 G453 G454 G458 G459	4359
Sleep disorders	G470 G471 G472 G473 G474 G478 G479	3479
Mononeuropathy, nerve root and plexus disorders of lower limbs	G543 G544 G541 G570 G571 G572 G573 G574 G575 G576 G578 G579	3550 3551 3555 3556 3558
Polyneuropathy	G600 G602 G603 G608 G609 G610 G618 G619 G620 G621 G622 G628 G629 G631 G632 G633 G636	
Myoneural disorders	G700 G702 G708 G709 G731	35809
Primary disorders of muscles	G710 G711 G712 G713 G718 G719	3593 3594

Paralytic syndromes affecting lower limbs	G800 G801 G802 G808 G809 G810 G811 G819 G821 G822 G824 G825 G831 G833 G834	3421 3429 34300 3431 3439 3441 3446 3449
Hydrocephalus	G910 G911 G912 G913 G918 G919	3314
Spinal cord disease	G950 G951 G952 G958 G959	3360 3369 3352
Diseases of the circulatory system		
Essential hypertension	I10	4019
Secondary hypertension	I110 I119 I120 I129 I130 I131 I132 I139 I150 I151 I152 I159	4039
All hypertensive disease	I10 I110 I119 I120 I129 I130 I131 I132 I139 I150 I151 I152 I159	4019 4039
All valve disease	I340 I341 I342 I348 I349 I350 I351 I352 I358 I359 I360 I361 I368 I369 I370 I371	3940 3942 3949 4240 4241
Angina	I200 I201 I208 I209	4139
Myocardial Infarction	I210 I211 I212 I213 I214 I219 I256 I220 I221 I228 I229 I252	4109 4129
Atherosclerotic heart disease	I251	
Heart Failure	I500 I501 I509	4280 4281
Pulmonary embolism	I260 I269	4151
Arterial thromboembolism	I740 I741 I742 I743 I744 I745 I748 I749	4442 4448
Venous thromboembolism	I820 I822 I823 I828 I829 I81	4538
Thromboembolic Stroke	I630 I631 I632 I633 I634 I635 I636 I638 I639	
Hemorrhagic Stroke	I610 I611 I612 I613 I614 I615 I616 I618 I619 I620 I621 I629	
Cardiomyopathy	I420 I421 I422 I424 I425 I426 I427 I428 I429	4254
Atrial Fibrillation		4273
All arrhythmia not atrial fibrillation	I440 I441 I442 I443 I444 I446 I447 I450 I451 I452 I453 I454 I455 I456 I458 I459 I470 I471 I472 I479 I490 I491 I492 I493 I494 I495 I498 I499	4270 4271 4272 4274 4276 4278 4279
All arrhythmia inc. atrial fibrillation	I440 I441 I442 I443 I444 I446 I447 I450 I451 I452 I453 I454 I455 I456 I458 I459 I470 I471 I472 I479 I490 I491 I492 I493 I494 I495 I498 I499	4270 4271 4272 4274 4276 4278 4279
Aneurysms	I711 I712 I713 I714 I715 I716 I718 I719 I720 I721 I722 I723 I724 I725 I728 I729 I254	4141 4373 4410 4411 4414 4416 4423 4429
Chronic rheumatic heart disease	I050 I051 I052 I058 I059 I060 I061 I062 I069 I070 I071 I078 I080 I081 I082 I083 I088 I089 I091 I098 I099	3940 3942 3949 3969 3989
Diseases of the respiratory system		
Allergic Rhinitis	J300 J301 J302 J303 J304	4778 4779
Chronic rhinitis, nasopharyngitis and pharyngitis	J310 J311 J312	4720 4721 4722
Chronic sinusitis	J320 J321 J322 J323 J324 J328 J329	4730 4731 4732 4733 4739
Chronic Laryngitis	J370	4760
Chronic Bronchitis	J410 J411 J42	4910 4912 4918
COPD	J430 J431 J432 J438 J439 J440 J441 J448 J449	4929

Asthma	J450 J451 J458 J459 J46	49309 49319 49390 49399
Bronchiectasis	J47	4949
Occupational lung disease	J60 J61 J62 J628 J634 J638 J64 J660 J668 J670 J672 J678 J679 J680 J683 J689	5019 4952
Interstitial pulmonary disease	J848 J849 J703 J704 J458 J459 J46	5159
Chronic suppurative and necrotic conditions of lower respiratory tract	J850 J851 J852 J853 J860 J869	5109
Diseases of the digestive system		
GORD	K210 K219 K227	53010 53011 53019
Achalasia	K220	5300
Esophageal obstruction	K221	
Esophageal dyskinesia	K224	5305
Diverticulum of esophagus	K225	
Peptic ulcer disease	K221 K250 K251 K252 K253 K254 K255 K256 K257 K259 K260 K261 K262 K263 K264 K265 K266 K267 K269 K270 K273 K274 K275 K276 K277 K279 K280 K283 K284 K285 K286 K287 K289	5302 5305 5310 5311 5314 5315 5317 5319 5320 5321 5323 5324 5325 5326 5327 5329 5334 5339 5343 5344 5349
Gastritis and duodenitis	K290 K291 K292 K293 K294 K295 K296 K297 K298	5350 5351 5353 5354 5355 5356
Functional Intestinal Disorders	K580 K589 K30 K590	5368 5369
Appendicitis	K350 K351 K352 K353 K358 K359 K36 K37	5400 5401 5409 5419 5429
Inguinal Hernia	K400 K401 K402 K403 K404 K409	5500 5501 5509
Femoral Hernia	K410 K412 K413 K414 K419	5520 5530
Umbilical hernia	K420 K421 K429	5521 5531
Ventral hernia	K430 K431 K439	5522 5532
Diaphragmatic hernia	K440 K441 K449	5523 5533
Other abdominal hernia	K450 K451 K458 K460 K461 K469	5528 5538 5539
Regional Enteritis	K500 K501 K508 K509	5550 5551 5552 5559
Ulcerative Colitis	K510 K511 K512 K513 K514 K515 K518 K519	
Angiodysplasia of colon	K552	
Diverticular disease of intestine	K570 K571 K572 K573 K574 K575 K578 K579	5620 5621
Fatty Liver Disease	K760	
Chronic cholecystitis	K811	
Chronic Pancreatitis	K860 K861	5771
Coeliac disease	K900	5790

Diseases of the musculoskeletal system and connective tissue		
Rheumatoid arthritis	M0500 M0509 M0530 M0580 M0582 M0583 M0584 M0586 M0587 M0588 M0590 M0591 M0592 M0593 M0594 M0595 M0596 M0597 M0598 M0599 M0600 M0601 M0602 M0603 M0604 M0605 M0606 M0607 M0608 M0609 M0640 M0641 M0643 M0645 M0646 M0647 M0649 M0680 M0681 M0682 M0685 M0686 M0687 M0688 M0689 M0690 M0691 M0692 M0693 M0694 M0695 M0696 M0697 M0698 M0699	71400 71401 71403 71404 71405 71406 71409 71416
Crystal Arthopathies	M1000 M1002 M1003 M1004 M1006 M1007 M1008 M1009 M1090 M1092 M1093 M1094 M1095 M1096 M1097 M1099 M1116 M1120 M1121 M1123 M1124 M1125 M1126 M1127 M1128 M1180 M1181 M1182 M1183 M1186 M1190 M1191 M1192 M1193 M1194 M1196 M1197 M1199	2749
Arthroses	M1500 M1501 M1502 M1503 M1504 M1508 M1509 M160 M161 M162 M163 M164 M165 M166 M167 M169 M170 M171 M172 M173 M174 M175 M179 M180 M181 M182 M183 M185 M189 M1900 M1901 M1902 M1903 M1904 M1905 M1906 M1907 M1908 M1910 M1911 M1912 M1913 M1914 M1915 M1916 M1917 M1918 M1919 M1921 M1922 M1923 M1924 M1925 M1927 M1981 M1984 M1986 M1987 M1988 M1989 M1990 M1991 M1992 M1993 M1994 M1995 M1996 M1997 M1998 M1999	71514 71515 71516 71526 71527 71531 71533 71534 71535 71536 71537 7158 7159 71650 71656 71659 71666 71691 71694 71695 71696 71697 71699
Meniscal & Ligament damage of the knee		2320 2321 2322 2323 2324 2325 2326 2327 2329 2330 2331 2332 2333 2334 2335 2336 2337 2339 2340 2341 2342 2343 2344 2345 2346 2349 2350 2351 2352 2353 2354 2355 2356 2359 2361 2362 2363 2366 2369 2380 2381 2382 2383 2384 2385 2386 2387 2389 2390 2391 2392 2393 2394 2396 2399 7170 7171 7172 7173 7174 7175 7176 7177 7178 7179
Dorsalgia	M5402 M5405 M5406 M5407 M5409 M5410 M5412 M5413 M5414 M5415 M5416 M5417 M5418 M5419 M5420 M5421 M522 M5423 M5424 M5428 M5429 M5430 M5432 M5435 M5436 M5437 M5438 M5439 M5440 M5444 M5445 M5446 M5447 M5448 M5449 M5450	7231 7234 7235 7238 7240 7242 7243 7244 7245 7246 7247 7248 7249

Dorsopathies	M5452 M5453 M5454 M5455 M5456 M5457 M5458 M5459 M5460 M5463 M5464 M5465 M5466 M5467 M5468 M5469 M5480 M5482 M5483 M5484 M5485 M5486 M5487 M5488 M5489 M5490 M5491 M5492 M5493 M5494 M5495 M5496 M5497 M5498 M5499 M5402 M5405 M5406 M5407 M5409 M5410 M5412 M5413 M5414 M5415 M5416 M5417 M5418 M5419 M5420 M5421 M522 M5423 M5424 M5428 M5429 M5430 M5432 M5435 M5436 M5437 M5438 M5439 M5440 M5444 M5445 M5446 M5447 M5448 M5449 M5450 M5452 M5453 M5454 M5455 M5456 M5457 M5458 M5459 M5460 M5463 M5464 M5465 M5466 M5467 M5468 M5469 M5480 M5482 M5483 M5484 M5485 M5486 M5487 M5488 M5489 M5490 M5491 M5492 M5493 M5494 M5495 M5496 M5497 M5498 M5499	7231 7234 7235 7238 7240 7242 7243 7244 7245 7246 7247 7248 7249
Shoulder lesions	M750 M751 M752 M753 M754 M755	7260 7261 7262
Osteoporosis	M8008 M8048 M8049 M8058 M8080 M8081 M8086 M8088 M8089 M8090 M8091 M8092 M8093 M8094 M8095 M8096 M8097 M8098 M8099 M8100 M8105 M8108 M8109 M8110 M8120 M8129 M8140 M8144 M8148 M8149 M8155 M8159 M8167 M8168 M8180 M8185 M8187 M8188 M8189 M8190 M8191 M8193 M8194 M8195 M8196 M8197 M8198 M8199	73305 73309
Diseases of the genitourinary system		
Chronic renal failure	N180 N181 N182 N183 N184 N185 N188 N189 N19	5859
Urolithiasis	N200 N201 N202 N209 N210 N211 N218 N219	5920 5921 5929
Cystitis	N300 N301 N302 N303 N304 N308 N309	5950 5951 5952 5953 5958 5959
Neuromuscular dysfunction of bladder	N310 N311 N312 N318 N319	
Hyperplasia of prostate	N40	60099
Chronic Prostatitis	N411	6011
Hydrocele	N433	6039
Benign mammary dysplasia	N600 N601 N602 N603 N604 N608 N609	6100 6101 6102 6103 6104 6108 6109
Inflammatory disorders of breast	N61	6110
Dysplasia of cervix uteri	N61	6110
Chronic salpingitis and oophoritis	N701	6141
Inflammatory disease of uterus, except cervix	N711	6151
Inflammatory disease of the cervix	N72	6160
Endometriosis	N800 N801 N802 N803 N804 N805 N806 N808 N809	6170 6171 6172 6173 6176 6178 6179
Female genital Prolapse	N810 N811 N812 N813 N814 N815 N816 N818 N819	6180 6181 6184 6186 6187 6188 6189

Fistulae involving female genital tract	N820 N821 N822 N823 N824 N825 N828 N829 N810	6190 6191 6192 6198 6199
Excessive, frequent and irregular menstruation	N920 N921 N922 N923 N924 N925 N926	6261 6262 6264 6265 6266 6267 6268 6269
Female infertility	N970 N971 N972 N973 N974 N978 N979	6280 6282 6283 6284 6288 6289
Male infertility	N46	6069
Diseases of the ear & mastoid		
Chronic diseases of the middle ear and mastoid process	N652 N653 N654 N661 N662 N663 N701 N702 N708 N709 N731	3811 3812 3813 3821 3822 3823 3831 3850 3851 3852 3853
Inner ear disorders	H800 H801 H802 H808 H809 H810 H811 H812 H813 H814 H818 H819 H830 H831 H832 H833 H838 H839	3860 3861 3863 3869 3872 3879 388
Hearing loss	H900 H901 H902 H903 H904 H905 H906 H907 H908 H911 H912 H913 H918 H919	3890 3891 3892 3899
Disorders of the eyes		
Cataracts	H250 H251 H252 H258 H259 H260 H261 H262 H263 H264 H268 H269	3662 3665 3668 3669
Disorders of choroid and retina	H300 H301 H302 H308 H309 H310 H311 H312 H313 H314 H318 H319 H330 H331 H332 H333 H334 H335 H340 H341 H342 H348 H349 H350 H351 H352 H353 H354 H355 H356 H357 H358 H359 H360 H368	3632 3638
Glaucoma	H400 H401 H402 H403 H404 H405 H406 H408 H409 H428	3650 3651 3652 3655 3656 3659
Disorders of ocular muscles & binocular movement	H490 H491 H492 H493 H494 H498 H499 H500 H501 H502 H503 H504 H505 H506 H508 H509 H510 H511 H512 H518 H519	3780 3781 3784 3785 3788 3789
Visual disturbances and blindness	H530 H531 H532 H533 H534 H535 H538 H539 H540 H541 H542 H544 H545 H546 H547 H549	3682 3684 3688 3689 3690 3696 3697 3699

Supplementary Table 3. Joint effects of anemia and C-creative protein/chronic conditions on dementia risk.

**Joint Exposure**		**Number of Participants**	**Dementia β (95% CI)**
CRP level	Anemia		
Low	No	95,642	Reference
High	No	94,253	0.96 (0.90, 1.03)
Low	Yes	3,267	1.49 (1.23, 1.80)
High	Yes	4,503	1.89 (1.60, 2.22)
		p-interaction = 0.032	
Chronic conditions	Anemia		
No	No	106,731	Reference
Yes	No	92,263	1.44 (1.34, 1.53)
No	Yes	2,956	1.54 (1.21, 1.95)
Yes	Yes	5,253	2.57 (2.21, 2.98)
		p-interaction = 0.290	

CI, confidence interval; CRP, C-creative protein.

Model adjusted for age, sex, education, race, Townsend deprivation index, smoking status, alcohol consumption status, body mass index, regular physical activity, active social connection, *apolipoprotein E* epsilon 4, as well as CRP level and chronic conditions, if applicable.

Supplementary Table 4. Hazard ratios (HRs), 50th Percentile Differences (PDs) in Years, and 95% confidence intervals (CIs) of dementia in relation to anemia: after multiple imputations for covariates.

**Anemia**	**No. of subjects**	**No. of cases**	**Cox Regression**		**Laplace Regression**	
			HR (95% CI)†	HR (95% CI)‡	50th PDs (95% CI)†	50th PDs (95% CI)‡
No	198,994	5,828	Reference (1.00)	Reference (1.00)	Reference (0)	Reference (0)
Yes	8,209	444	1.93 (1.76, 2.13)*	1.56 (1.41, 1.72)*	-0.19 (-0.23, -0.16)*	-1.87 (-2.21, -1.52)*

† Adjusted for age, sex, and education.

‡ Adjusted for age, sex, education, race, Townsend deprivation index, smoking status, alcohol consumption status, body mass index, regular physical activity, active social connection, *apolipoprotein E* epsilon 4, C-creative protein, and chronic conditions.

* $p < 0.05$

Supplementary Table 5. Hazard ratios (HRs), 50th Percentile Differences (PDs) in Years, and 95% confidence intervals (CIs) of dementia in relation to anemia: further adjusted for eGFR.

Anemia	No. of subjects	No. of cases	Cox Regression		Laplace Regression	
			HR (95% CI)†	HR (95% CI)‡	50th PDs (95% CI)†	50th PDs (95% CI)‡
No	198,994	5,828	Reference (1.00)	Reference (1.00)	Reference (0)	Reference (0)
Yes	8,209	444	1.93 (1.76, 2.13)*	1.62 (1.43, 1.84)*	-0.19 (-0.23, -0.16)*	-1.61 (-2.05, -1.18)*

† Adjusted for age, sex, and education.

‡ Adjusted for age, sex, education, race, Townsend deprivation index, smoking status, alcohol consumption status, body mass index, regular physical activity, active social connection, *apolipoprotein E* epsilon 4, C-creative protein, eGFR, and chronic conditions.

* $p < 0.05$

Supplementary Table 6. Hazard ratios (HRs), 50th Percentile Differences (PDs) in Years, and 95% confidence intervals (CIs) of dementia in relation to anemia: stratified by smoking status.

Anemia	No. of subjects	No. of cases	Cox Regression		Laplace Regression	
			HR (95% CI)†	HR (95% CI)‡	50th PDs (95% CI)†	50th PDs (95% CI)‡
**Never**						
No	98,994	2,636	Reference (1.00)	Reference (1.00)	Reference (0)	Reference (0)
Yes	3,876	199	1.97 (1.70, 2.28)*	1.85 (1.54, 2.22)*	-0.16 (-0.21, -0.12)*	-1.84 (-2.50, -1.18)*
**Former**						
No	82,387	2,591	Reference (1.00)	Reference (1.00)	Reference (0)	Reference (0)
Yes	3,729	203	1.81 (1.57, 2.10)*	1.62 (1.35,1.95)*	-0.19 (-0.25, -0.14)*	-1.29 (-1.94, -0.65)*
**Current smoker**						
No	16,423	545	Reference (1.00)	Reference (1.00)	Reference (0)	Reference (0)
Yes	536	35	2.21 (1.55, 3.14)*	1.78 (1.09, 2,91)*	-0.54 (-0.76, -0.32)*	-1.57 (-3.31, 0.17)

† Adjusted for age, sex, and education.

‡ Adjusted for age, sex, education, race, Townsend deprivation index, alcohol consumption status, body mass index, regular physical activity, active social connection, *apolipoprotein E* epsilon 4, C-creative protein, and chronic conditions.

* $p < 0.05$

The interaction between anemia and smoking status on incident dementia is not significant (*p*-interaction = 0.160).

Supplementary Table 7. Hazard ratios (HRs), 50th Percentile Differences (PDs) in Years, and 95% confidence intervals (CIs) of dementia in relation to anemia: stratified by levels of physical activity.

**Anemia**	**No. of subjects**	**No. of cases**	**Cox Regression**		**Laplace Regression**	
			HR (95% CI)†	HR (95% CI)‡	50th PDs (95% CI)†	50th PDs (95% CI)‡
**Irregular physical activity**						
No	50,712	1,531	Reference (1.00)	Reference (1.00)	Reference (0)	Reference (0)
Yes	2,533	140	1.91 (1.60, 2.28)*	1.50 (1.22, 1.86)*	-0.27 (-0.35, -0.20)*	-1.34 (-2.08, -0.60)*
**Regular physical activity**						
No	128,100	3,372	Reference (1.00)	Reference (1.00)	Reference (0)	Reference (0)
Yes	4,464	214	1.85 (1.60, 2.12)*	1.60 (1.36, 1.87)*	-0.13 (-0.18, -0.09)*	-1.58 (-2.12, -1.04)*

† Adjusted for age, sex, and education.

‡ Adjusted for age, sex, education, race, Townsend deprivation index, smoking status, alcohol consumption status, body mass index, active social connection, *apolipoprotein E* epsilon 4, C-creative protein, and chronic conditions.

*$p < 0.05$

The interaction between anemia and levels of physical activity on incident dementia is not significant (*p*-interaction = 0.873).

Supplementary Table 8. Hazard ratios (HRs) and 95% confidence intervals (CIs) of dementia in relation to anemia: stratified by levels of eGFR.

Anemia	No. of subjects	No. of cases	Cox Regression	
			HR (95% CI)[†]	HR (95% CI)[‡]
**Normal kidney function**				
No	182,050	5,183	Reference (1.00)	Reference (1.00)
Yes	6,292	307	1.91 (1.60, 2.28)[*]	1.62 (1.40, 1.86)[*]
**Impaired kidney function**				
No	7,964	387	Reference (1.00)	Reference (1.00)
Yes	1,525	114	1.85 (1.60, 2.12)[*]	1.57 (1.19, 2.08)[*]

[†] Adjusted for age, sex, and education.

[‡] Adjusted for age, sex, education, race, Townsend deprivation index, smoking status, alcohol consumption status, body mass index, regular physical activity, active social connection, *apolipoprotein E* epsilon 4, C-creative protein, and chronic conditions.

[*] $p < 0.05$

Supplementary Table 9. Hazard ratios (HRs) and 95% confidence intervals (CIs) of dementia in relation to anemia: using competing risk model (the Fine-Gray model) with death as competing events.

**Anemia**	**No. of subjects**	**No. of cases**	**HR (95% CI)**[†]	**HR (95% CI)**[‡]
No	198,994	5,828	Reference (1.00)	Reference (1.00)
Yes	8,209	444	2.34 (2.24, 2.45)*	1.97 (1.86, 2.09)*

[†] Adjusted for age, sex, and education.

[‡] Adjusted for age, sex, education, race, Townsend deprivation index, smoking status, alcohol consumption status, body mass index, regular physical activity, active social connection, *apolipoprotein E* epsilon 4, C-creative protein, and chronic conditions.

*$p < 0.05$

Supplementary Table 10. Hazard ratios (HRs), 50th Percentile Differences (PDs) in Years, and 95% confidence intervals (CIs) of dementia in relation to anemia: after excluding incident dementia within the first five years of follow-up.

**Anemia**	**No. of subjects**	**No. of cases**	**Cox Regression**		**Laplace Regression**	
			HR (95% CI)†	HR (95% CI)‡	50th PDs (95% CI)†	50th PDs (95% CI)‡
No	198,475	5,309	Reference (1.00)	Reference (1.00)	Reference (0)	Reference (0)
Yes	8,142	377	1.79 (1.60, 1.99)*	1.49 (1.30, 1.71)*	-0.18 (-0.21, -0.15)*	-1.40 (-1.80, -1.00)*

† Adjusted for age, sex, and education.

‡ Adjusted for age, sex, education, race, Townsend deprivation index, smoking status, alcohol consumption status, body mass index, regular physical activity, active social connection, *apolipoprotein E* epsilon 4, C-creative protein, and chronic conditions.

* $p < 0.05$
